# Primary intestinal lymphangiectasia (Waldmann's disease)

**DOI:** 10.1186/1750-1172-3-5

**Published:** 2008-02-22

**Authors:** Stéphane Vignes, Jérôme Bellanger

**Affiliations:** 1 Department of Lymphology, Centre de référence des maladies vasculaires rares, Hôpital Cognacq-Jay, 15, rue Eugène Millon, 75015 Paris, France; 2 Department of Gastroenterology and Nutrition, Hôpital Saint-Antoine, AP-HP, 184 rue du Faubourg Saint-Antoine, 75571 Paris Cedex 12, France

## Abstract

Primary intestinal lymphangiectasia (PIL) is a rare disorder characterized by dilated intestinal lacteals resulting in lymph leakage into the small bowel lumen and responsible for protein-losing enteropathy leading to lymphopenia, hypoalbuminemia and hypogammaglobulinemia. PIL is generally diagnosed before 3 years of age but may be diagnosed in older patients. Prevalence is unknown. The main symptom is predominantly bilateral lower limb edema. Edema may be moderate to severe with anasarca and includes pleural effusion, pericarditis or chylous ascites. Fatigue, abdominal pain, weight loss, inability to gain weight, moderate diarrhea or fat-soluble vitamin deficiencies due to malabsorption may also be present. In some patients, limb lymphedema is associated with PIL and is difficult to distinguish lymphedema from edema. Exsudative enteropathy is confirmed by the elevated 24-h stool α1-antitrypsin clearance. Etiology remains unknown. Very rare familial cases of PIL have been reported. Diagnosis is confirmed by endoscopic observation of intestinal lymphangiectasia with the corresponding histology of intestinal biopsy specimens. Videocapsule endoscopy may be useful when endoscopic findings are not contributive. Differential diagnosis includes constrictive pericarditis, intestinal lymphoma, Whipple's disease, Crohn's disease, intestinal tuberculosis, sarcoidosis or systemic sclerosis. Several B-cell lymphomas confined to the gastrointestinal tract (stomach, jejunum, midgut, ileum) or with extra-intestinal localizations were reported in PIL patients. A low-fat diet associated with medium-chain triglyceride supplementation is the cornerstone of PIL medical management. The absence of fat in the diet prevents chyle engorgement of the intestinal lymphatic vessels thereby preventing their rupture with its ensuing lymph loss. Medium-chain triglycerides are absorbed directly into the portal venous circulation and avoid lacteal overloading. Other inconsistently effective treatments have been proposed for PIL patients, such as antiplasmin, octreotide or corticosteroids. Surgical small-bowel resection is useful in the rare cases with segmental and localized intestinal lymphangiectasia. The need for dietary control appears to be permanent, because clinical and biochemical findings reappear after low-fat diet withdrawal. PIL outcome may be severe even life-threatening when malignant complications or serous effusion(s) occur.

## Disease name and synonyms

Primary intestinal lymphangiectasia (PIL).

Waldmann's disease.

## History and definition

In 1961, Waldmann *et al*. described the first 18 cases of "idiopathic hypercatabolic hypoproteinemia" [1]. These patients had edema associated with hypoproteinemia, low serum albumin and gammaglobulin levels. The total exchangeable albumin pool, assessed with radio-labeled ^131^I-albumin, was low in all patients. Daily fecal excretion of ^131^I was twice the highest value obtained in controls. Microscope examination of the small intestine biopsies showed variable degrees of dilation of the lymph vessels in the mucosa and submucosa. The authors also proposed the term "intestinal lymphangiectasia".

## Epidemiology

The prevalence of clinically overt PIL is unknown. However, PIL can be asymptomatic; it primarily affects children (generally diagnosed before 3 years of age) and young adults but may be diagnosed later in adults [2,3]. Very rare familial forms of Waldmann's disease have been reported [1,4].

## Clinical description

### A. Edema

Peripheral edema of variable degree, usually symmetrical, from moderate (lower limb edema) to severe, including the face and external genitalia, is the main clinical feature, which accounts for 95% of PIL clinical manifestations [4]. Moderate serous effusions (pleural effusion, pericarditis, chylous ascites) are common, and life-threatening anasarca may occur rarely throughout the course of the disease [1,5]. The edema is pitting because the oncotic pressure is low due to hypoalbuminemia resulting from exsudative enteropathy. PIL may be suspected at birth or during pregnancy based on ultrasonography images, which can detect fetal ascites or lower limb lymphedema [6].

### B. Childhood particularities

In children, PIL is generally diagnosed before 3 years of age [5,7,8] and may be complicated by fatigue, abdominal pain, nausea, vomiting and weight loss, inability to gain weight and growth retardation [5]. Malabsorption may cause fat-soluble vitamin deficiencies and hypocalcemia leading to convulsions [5].

### C. Other clinical findings

**Figure 1:**
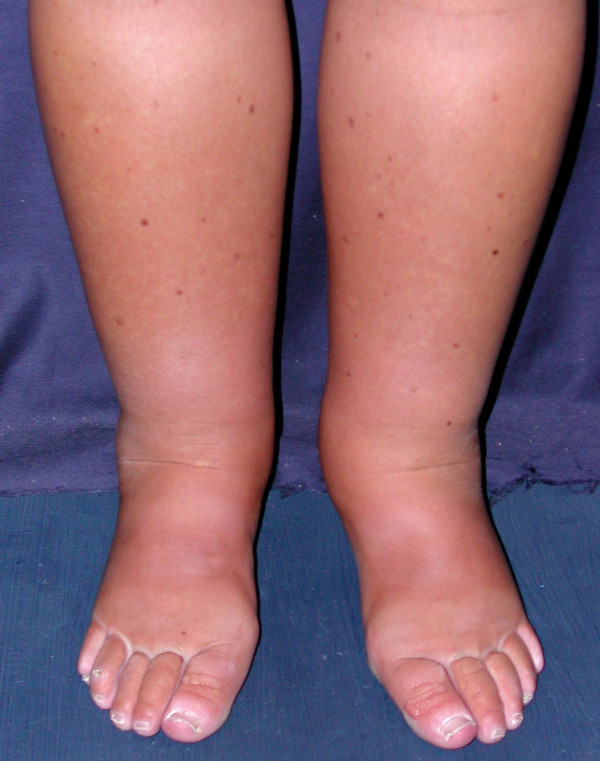
**A 23-year-old woman with PIL since infancy.** Note the bilateral lower limb lymphedema, with accentuation of the dorsal flexion folds of the toes.

- **Lymphedema **is a rare disorder which is usually not associated with another disease, but it may be associated with intestinal lymphangiectasia. Clinical features of lymphedema are specific (Figure 1). Lymphedema is less pitting than edema due to hypoproteinemia, and is localized to the lower limbs (foot, ankle, calf, rarely thigh) and predominantly bilateral. Upper limb with hand and forearm involvement, lymphedema of breast and external genitalia (with skin thickening) may also be present [9]. In most patients with lymphedema, edema as a consequence of hypoproprotidemia has also been observed. The two types of edema are not always easily distinguished. Stemmer's sign is an important element to confirm the diagnosis and differentiate lymphedema from edema: *i.e*., it is impossible to lift and wrinkle the dorsal skin on the second toe because of skin-thickening as the result of fibrosis.

- Moderate or discontinuous **diarrhea **is the main digestive symptom [10].

- An **abdominal mass **was found in the epigastrium and right upper quadrant of a 12-year-old girl. Ultrasonography failed to confirm an abdominal mass but the mass was attributed to small bowel edema [11].

- A **malabsorption syndrome **may be encountered in the elderly [12].

- **Mechanical ileus **of the small intestine caused by localized edema leading to intestinal wall-thickening and lumen diminution. It may be visualized as a segmental mass requiring resection (histological findings: dilation of submucosal lymphatics, extensive pseudocysts in the jejunal wall, intramural lymph edema, secondary bleeding, tight stenosis of the jejunal lumen) [13].

- **Chylous reflux **into the skin of the right flank resembles lymphangioma circumscriptum with multiple dome-shaped vesicles filled with milky-white fluid, which is discharged into the surrounding skin [14].

- Chyle can also backflow into the skin of the lower limb, perineum or external genitalia [15].

- An **association with celiac disease **has been reported in children [16].

- **Iron deficiency with anemia **occurs secondary to chronic blood loss resulting from non-specific multiple ulcers in the small intestine [17].

- **Necrolytic migratory erythema **can be seen [18].

- **Recurrent hemolytic uremic syndrom**e has been described [19].

- **Osteomalacia **resulting from vitamin D deficiency was reported in a 63-year-old woman [20].

- It remains open to debate whether **recurrent gastrointestinal bleeding **is indeed a manifestation of PIL [21].

### D. Syndromes associated with intestinal lymphangiectasia

First described in 1964 by Samman and White, the yellow nail syndrome is a very rare condition which may be associated with PIL. Dystrophic yellow nails with ridging and loss of lunula on the hands are associated with lymphedema and pleural effusions [22].

Five syndromes are associated with intestinal lymphangiectasia: von Recklinghausen, Turner (X0) or Noonan, Klippel-Trenaunay and Hennekam [23]. In general, these syndromes are easily distinguishable by the presence of facial abnormalities (Turner, Noonan, Hennekam), mental retardation (Hennekam, Noonan), seizures (Hennekam), severe limb and/or face lymphedema (Hennekam), neurofibromas and other tumors (von Recklinghausen), and hemihypertophy of the limbs associated with vascular malformations (Klippel-Trenaunay).

## Etiology and pathogenesis

To date, PIL etiology is unknown. Intestinal lymphangiectasia is responsible for lymph leakage into the bowel lumen, which leads to hypoalbuminemia and lymphopenia. Edema is the consequence of hypoprotidemia with decreased oncotic pressure. Several genes, such as *VEGFR3 *(vascular endothelial growth factor receptor 3), prospero-related homeobox-transcriptional factor *PROX1*, forkhead transcriptional factor *FOXC2 *and *SOX18 *are implicated in the development of the lymphatic system. In a recent paper, Hokari *et al*. reported inconsistently changed expressions of regulatory molecules for lymphangiogenesis in the duodenal mucosa of PIL patients [24].

## Diagnosis and diagnostic methods

**Figure 2:**
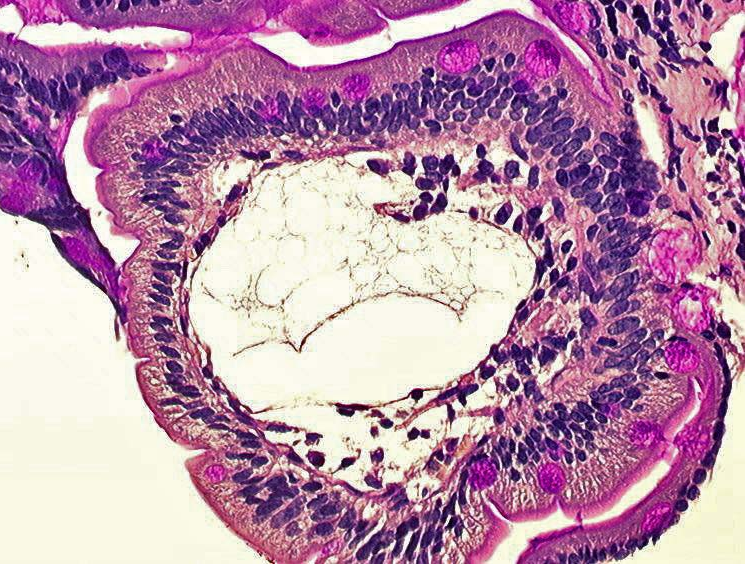
Duodenal biopsy: note the markedly dilated lymphatic ducts (periodic acid Schiff staining).

**Figure 3:**
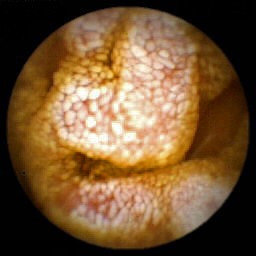
Capsule endoscopy (M2A, Given Imaging, Yoqneam, Israel): note the edematous aspect of the jejunum mucosa with whitish, swollen villi.

PIL diagnosis is confirmed by the presence of intestinal lymphangiectasia based on endoscopic findings with the corresponding histology of intestinal biopsy specimens (Figure 2). Macroscopic abnormalities are usually obvious with creamy yellow of jejunal villi corresponding to marked dilation of the lymphatics within the intestinal mucosa. The density of lymphangiectasia varies and their size ranges from mm to cm. Histological examination of duodenum-jejunum and ileum biopsies confirms the presence of lacteal juice, dilated mucosal (from moderate to severe) and submucosal lymphatic vessels (and also in the serosa) with polyclonal normal plasma cells. Intestinal lymphatics may be dilated in many villi or only a few. Intestinal abnormalities may be slight; small bowel mucosa also appears edematous but not creamy. That neither villous atrophy nor microorganisms are found in biopsies is underlined in pathology reports. Endoscopy may be negative when intestinal lesions are segmental or localized. In such cases, videocapsule endoscopy, easier to use than enteroscopy, is a useful tool to detect the presence of intestinal lymphangiectasia and to specify its localization (Figure 3) [25-27]. Videocapsule endoscopy is also feasible to appreciate the extent of lymphangiectasia in children [28].

Indirect biological abnormalities are suggesting of PIL, such as hypoproteinemia, hypoalbuminemia, hypogammaglobulinemia with low IgG, IgA and IgM levels or lymphocytopenia. Exsudative enteropathy is confirmed by the high 24-h stool α1-antitrypsin clearance due to enteric protein loss. Functional absorption tests, *e.g*., such as D-xylose test results are normal in PIL.

### Complementary examinations

Although various methods have been proposed to investigate PIL, none of them can replace histological examination of biopsies to confirm the diagnosis.

#### A. Albumin scintigraphy

^99m^Technetium-labeled human serum albumin (^99m^Tc-HSA) scintigraphy may show marked enhancement in the bowel, which indicates protein leakage into this region [23]. Serial scanning for up to 24 h is required to detect protein loss from the gut, perhaps because of the intermittent nature of protein loss. ^99m^Tc-HSA scintigraphy has high sensitivity and is able to identify the site of protein loss (large and/or small bowel localization, stomach) [29]. The α1-antitrypsin method has replaced ^99m^Tc-HSA scintigraphy, which is more costly, less readily available and also uses a human product potentially carrying an infectious risk.

#### B. Ultrasound

Indirect features may suggest PIL in children and adults. Ultrasonographic findings may show dilation of the intestinal loops, regular and diffuse thickening of the walls, plical hypertrophy and severe mesenteric edema and, in some cases, ascites [30,31].

#### C. Computed tomography (CT) scans

Axial abdominal CT images are obtained with oral and intravenous contrast medium enhancement. CT appearance of PIL is similar in adults and children. Typically, there is diffuse, nodular, small bowel wall-thickening and edema, which are a consequence of the dilated lymphatics within the villi, along with some degree of small bowel dilation, with, in few cases, a "halo sign" due to swelling and edema [32-34]. CT may be useful to identify localized intestinal lymphangiectasia [35].

#### D. Lymphoscintigraphy

Lymphoscintigraphy is an effective tool for identifying abnormal lymphatic tree in the upper or lower limb and also to confirm limb lymphedema, when the limb images are abnormal. In limb lymphedema, isotope-uptake seen on the lymphogram shows an absence of visible lymph nodes which indicates either peripheral lymphatic obliteration or inability of the vessels to transport lymph up the limb through incompetent lymphatic vessels [36]. ^99m^Tc-radio-labeled rhenium sulfur colloid and albumin colloid are the two isotopes currently used in limb lymphoscintigraphy. At present, lymphoscintigraphy is not a routine and useful methodology for PIL diagnosis. Indeed, in one study, So *et al*. reported that intestinal lymphoscintigraphy (after subcutaneous injection of ^99m^Tc-antimony sulfide colloid into the two first toe spaces) only detected the presence of radioactivity in less than half of patients with histologically proven PIL [37].

### Immunological abnormalities

PIL patients have immunological abnormalities involving both the B-cell and T-cell lineages of the immune system. The B-cell defect is characterized by low immunoglobulin levels (IgG, IgA and IgM) and poor antibody responses [38-40]. The T-cell defect is characterized by lymphocytopenia, prolonged skin-allograft rejection and impaired *in vitro *proliferative responses to various stimulants (anti-CD3, anti-CD28) [41]. Furthermore, PIL patient's peripheral blood samples contain extremely low counts of CD4^+ ^T cells, especially naïve, CD45RA^+ ^CD62L^+^, while CD45RO^+ ^memory cells, are only moderately below normal. CD45RA^+ ^and CD45RO^+ ^CD8^+ ^T cells are moderately below normal [41].

## Differential diagnosis

The differential diagnosis is of special importance for subjects suspected of having PIL. Some secondary causes of intestinal lymphangiectasia have been identified as diseases responsible for anatomical or dynamic alterations of the lymphatic flow. Protein-losing enteropathies associated with intestinal lymphangiectasia may arise secondary to constrictive pericarditis [42-44], intestinal lymphoma [45,46], lymphenteric fistula [47], Whipple's disease [48], Crohn's disease [49], sarcoidosis [50], intestinal tuberculosis [51], systemic sclerosis [52], radiation and/or chemotherapy with retroperitoneal fibrosis [53], human immunodeficiency virus-related enteropathy [54] or the Fontan operation to treat cardiac malformations [55].

Many diseases are associated with excessive enteric loss of plasma proteins but do not include lymphatic lymphangiectasia, such as Menetrier's disease or inflammatory states of systemic lupus erythematosus in adults and children [56-59].

## Complications

### A. Malignant transformation

It is not clear whether the occurrence of malignancy, especially lymphomas, is fortuitous or related to PIL. Indeed, only a few lymphomas associated with PIL have been reported. Among 50 PIL patients reviewed, 3 had malignant lymphomas, that arose 3 to 25 years after the initial diagnosis [60]. The interval from PIL diagnosis to lymphoma diagnosis was even 39 and 40 years in a study [61]. Some lymphomas were confined to the gastrointestinal tract (stomach, jejunum, midgut, ileum), *i.e*. in PIL involved sites, while in other patients they were extra-intestinal (retroperitoneum, mediastinum, bone) [60-63]. In the well-described histological and immunological studies, only B-cell lymphomas (large cells, small cells, centroblastic, immunoblasts) were encountered [61]. Chemotherapy achieved regression of the exsudative enteropathy in only two of those previous cases [62,63].

Lymphoma may be related to PIL as a consequence of several mechanisms: (1) long-standing protein-losing enteropathy may be resolved after treatment (chemotherapy and/or radiotherapy) [62-64]; (2) lymphoma may cause secondary intestinal lymphangiectasia, (3) lymphoma in PIL could be associated with an immune deficiency. Indeed, a primary deficiency of B cells and/or helper T cells has been proposed in PIL, in combination with a secondary immune deficiency resulting from the loss of immunoglobulins and lymphocytes [39,40]. But no clear evidence of depressed intestinal immunity was found in PIL patients compared to those with other primary immunodeficiencies and having gut infections, like giardiasis.

### B. Cutaneous warts

PIL patients were reported to have diffuse and multiple cutaneous warts in association with lymphoma [65,66]. Widespread viral warts may be associated with a primary immune deficiency in PIL or secondary to lymphoma.

### C. Infections

Although PIL patients have moderate-to-severe hypogammaglobulinemia and lymphopenia, their risk of pyogenic bacterial infection is not significantly elevated and opportunistic infections are uncommon. Only one case of a severe infection with group G streptococcal empyema was reported [67] and another had cryptococcal meningitis [68].

### D. Gelatinous transformation of the bone marrow

It is an uncommon disorder characterized by the replacement of hematopoietic cells and adipocytes by amorphous extracellular material composed of acid mucopolysaccharides. The only reported case of a gelatinous transformation of the bone marrow in a PIL patient was attributed to the malnutrition resulting from malabsorption [69].

## Management including treatment

### A. Low-fat diet associated with medium-chain triglycerides

Low-fat diet associated with supplementary medium-chain triglycerides (MCT) is the cornerstone of PIL medical management [70]. It is likely that the absence of fat in the diet prevents engorgement of the intestinal lymphatics with chyle, thereby preventing their rupture with its ensuing protein and T-cell loss. MCT are directly absorbed into the portal venous circulation and thus provide nutrient fat but avoid lacteal engorgement. After a few weeks, this treatment may lead to reversal of clinical and biochemical signs (albuminemia, immunoglobulin levels and lymphocyte counts) [71]. In patients not responding to a low-fat diet, enteral nutritional therapy (elemental, semi-elemental and polymeric diets) may be required. In a few very severe cases, total parenteral nutrition is warranted [72]. The need for dietary control appears to be permanent, because clinical and biochemical findings reappear after low-fat diet withdrawal. Long-term PIL monitoring essentially concerns its predominant clinical manifestation (edema). Laboratory analyses (albuminemia, lymphocyte counts, immunoglobulin levels) are required when lower limb edema becomes more pronounced.

### B. Other treatments

In the literature, other treatments have been proposed to treat PIL. They can be used after or in combination with a low-fat diet associated with MCT supplementation. Their efficacy is variable and insufficiently evaluated.

#### 1) Antiplasmin

A few authors reported that PIL patients responded to tranexamic acid (1 g, 3 times a day) [73,74], but these responses were heterogeneous with only partial disease attenuation. It was hypothesized that increasing plasma fibrinolysis might enhance lymphatic permeability to plasma proteins. Under antiplasmin treatment, a lower percentage of T lymphocytes became normalized together with serum immunoglobulin values. In addition, the therapy resulted in the disappearance endoscopically observed duodenal lesions [73].

#### 2) Octreotide

In 1998, Ballinger and Farthing reported the efficacy of octreotide in one PIL patient [75]. To date, few publications have supported the contribution of octreotide in PIL. Octreotide (150–200 **μ**g, twice a day or the slow-release formulation) might lead to clinical, biochemical (albuminemia) and histological improvement [76,77]. The mechanism of action of the somatostatin analog on the gastrointestinal tract remains unclear. It has been shown that octreotide induces short-lasting splanchnic vasoconstriction in healthy volunteers and cirrhotic patients, and inhibits the absorption of triglycerides [78]. Furthermore, somatostatin inhibits thoracic lymph flow in dogs [79]. Octreotide may be useful in PIL patients in combination with a low-fat diet.

#### 3) Surgery

Small bowel resection is useful in the rare cases in which intestinal lymphangiectasia is segmental and localized (duodenum) [80,81]. Recurrent and/or bulky pleural effusions may lead to unilateral or bilateral surgical pleurectomy or talc pleurodesis [5].

#### 4) Corticosteroids

Steroids were prescribed to patients with intestinal lymphangiectasia secondary to inflammatory disease with variable efficacy [82] and also to systemic lupus erythematosus patients with protein-losing enteropathy [58]

#### 5) Albumin infusion

Albumin infusion is a symptomatic treatment proposed in patients with important serous effusion or uncomfortable lower limb edema. Repeated albumin intravenous supplementation may be useful to reduce edema and improve albuminemia but its efficacy is transient resulting from persistent lymph leakage into the bowel lumen.

## Prognosis and long-term outcome

PIL is a chronic debilitating disorder requiring constraining long-term dietary control based on a low-fat regimen associated with supplementary MCT. Lower limb edema is usually the main clinical manifestation but lymphedema may be associated. Lower limb lymphedema had its own particularities, including infectious complications (*e.g*., cellulitis) and requires specific long-term management (low-stretch bandage, manual lymph drainage, skin care, elastic hosiery) [83]. These two conditions deteriorate the quality of life (difficulty to put on shoes, unattractive aspect of leg). PIL outcome may be severe even life-threatening when malignant complications (lymphoma) or serous effusion(s) (pleural, pericardic) occur.
